# Surgery

**Published:** 1860-05

**Authors:** 


### Bi-Monthly Periscope.

### SURGERY.

1. CASES OF AMPUTATIONS WITH ACUPRESSURE. By *J. Y. Simpson, M.D.*, Professor of Medicine and Midwifery in the University of Edinburgh.—If after amputation of a limb, any British hospital surgeon at the present day were, for the purpose of arresting hemorrhage from the bleeding stump, to sear its raw surface over with red-hot irons, or besmear it with boiling pitch or turpentine, or apply to it potential caustics, the practice would be regarded as cruel and outrageous in the higest degree. Yet when Paré, in 1564, proposed in amputations the ligature of the bleeding vessels as a substitute for these cauteries and caustics, he was bitterly denounced and decried by most of his contemporaries; the French Parliament was petitioned to suppress the publication and dissemination of his doctrines; and the surgeons of the great Hospital at the Hôtel-Dieu of Paris (the city in which Paré himself lived and practiced) still continued, a century and a half afterwards, to use, as we learn from Dionis, in 1707, the vitriol button (a form of potential caustic) instead of the ligature, in all their amputations. And half a century later, Mr. Sharpe, Surgeon to Guy's Hospital, when writing in 1761, found it necessary, in his well-known work, entitled "A Critical Inquiry into the Present State of Surgery" in England, formally to advocate the employment of the ligature for the arrest of hemorrhage from wounded arteries, in preference to styptics or the cautery, on the ground that "it was not as yet universally practiced among surgeons residing in the more distant counties of our kingdom." (Erichsen's Surgery, p. 141.) In some semi-rude countries and communities, caustics are still the only hæmostatics employed after amputations. A few years ago my friend, Dr. Arthur Mitchell, when in Algeria, passed some time as a guest with a powerful Sheik in the Sahara. His host, who had been an officer of high rank in the army of Abd-el-Kadir, had his leg shattered by a French cannon-ball, and it was subsequently amputated by the surgeons of his country at the knee-joint. They stemmed the hemorrhage by applying hot pitch to the raw surface of the wound; and the stump itself was, as Dr. Mitchell assures me, an excellent and useful one.

If some correspondent of the *Times*, writing at the present hour from the camp of the army of Morocco, were to inform the English readers, professional and non-professional, that the African surgeons there, after amputating the damaged limbs of the soldiers wounded at Tetuan, deliberately placed five or six seton-threads between the surfaces of the flaps, and systematically attached and anchored these several seton-threads to little strangled pieces of flesh in the depths of the wounds, necessitating the seton-threads slowly to ulcerate, suppurate, and mortify through the points to which they were fixed before they were allowed to escape or be detached, perhaps many would, at first sight, deem such strange practices as characteristic of the "barbarous Moor." Yet, after all, this is only and truly what the enlightened surgeons of modern Europe do, when they stanch the hemorrhages after amputations by tying silken ligatures around the drawn-out and isolated ends of the bleeding arteries. For, assuredly, these silken ligatures, with the imbibed dead, and sometimes morbid, animal fluids decomposing in them, speedily and certainly become small irritating setons along the whole course of their tracks; and, as is known to every practical surgeon, and as has been especially pointed out by Bell, Crosse, Brodie, Pecot, Velpeau, Wise, and others, the portion of every artery strangled by the ligature ultimately sloughs off, and generally comes away in the loop of the ligature itself. Thus the author of the latest work published on surgery in the English language, namely, Professor Gross, of Philadelphia, when treating of this subject, observes: "The process by which the separation of the ligature is effected is worthy of inquiry. It is generally ascribed to ulcerative action, and this is undoubtedly true; but it is equally true that that portion of the artery immediately embraced by the ligature *mortifies, and comes away in the form of a slough.*" (System of Surgery, 1859, vol. i. p. 810.)

Union by the first intention, of the flaps of a stump after amputation, is certainly an object worthy of being aimed at by all possible means, more especially as the operation of amputation is usually performed in persons who have their constitutions damaged and shattered by previous disease or injury, and who are hence little capable of sustaining, without danger, any prolonged or severe local morbid action. For entire closure of the operation-wound by the first intention would transform a cure which usually lasts for many weeks into a cure of almost as many days; it would save the patient from the protracted suffering and distress produced by the continued dressings and discharges; and no doubt it would avert, in many instances, the dangers of pyæmic poisoning and surgical fever arising from the absorption of decomposing matters produced by local suppurations and sloughings in the depths of the wound.

But, for the reasons above adverted to, complete closure of an amputation-wound by the first intention is extremely rare, or, indeed, impossible, when ligatures are employed to secure the bleeding vessels. On the contrary, surgical wounds, under the most disadvantageous circumstances, readily enough unite by the first intention when arterial ligatures do not happen to be employed—as we see in the primary adhesion of the wound in hare-lip, despite the presence of the saliva; in the successful reunion of the revivified lips of vesico-vaginal fistulæ, despite the constant contact of urine; in the rapid closure of the wound in lithotomy; and in the success attending upon the whole class of operations included under the term of "plastic surgery"—a class of cases in which arterial ligatures are almost never employed.

Under the name of *acupressure*, or *acupression*, I some time ago proposed a plan of arresting hemorrhages in surgical wounds and operations by the employment of metallic pins or needles instead of ligatures. For the mode or modes of using these, I can only here refer to the descriptions which I have elsewhere published on the subject. (See *Edinburgh Medical Journal* for January 1, 1860, p. 645, and *Dublin Hospital Gazette* for January 2, 1860, p. 7.) They have this one very great advantage over ligatures, that the heads of the needles being left out on the cutaneous surface of the flap, the needles themselves can thus in every case be entirely withdrawn in forty or fifty hours, or as soon as the complete closure of the artery has been established, leaving no foreign body whatever in the flaps or walls of the wound to prevent their speedy and complete union. Besides, during the brief period that they are thus left in the wound, they are, as contrasted with organic ligatures, comparatively innocuous. For, in accordance with the general pathological law of the tolerance of animal structures for metallic bodies imbedded in them, metallic needles cause little or no irritation by their presence, as seen in the harmless retention for months or years of bullets, small-shot, pins, etc., in the body; in the relative innocuousness of long needles introduced and lodged for the purpose of acupuncture; in the non-irritating character of iron, silver, and other metallic threads, when used as surgical sutures; in the employment, by all our best surgeons, of metallic pins or needles in the union of hare-lip, where the whole aim and object of the operation is to secure and establish primary adhesion of the lips of the wound; and in the safe retention, during several days, of a metallic needle, passed through a fold of the peritoneum itself, in Rothmund's operation for the radical cure of hernia.

Granting, however, that acupressure by metallic needles is simpler and safer than the silken ligature, the great practical question remains—Is it actually sufficient for the closure of the arteries usually divided in surgical operations? Before publishing on the matter, I had experimented on the subject in the lower animals, and had shut up easily by it the carotid artery of the horse—the largest living vessel which was within my power to experiment upon. I had made also various experiments with it, in the way of amputation and other experiments, on the dead human subject—imitating the flow of blood by the injection of tepid water along the arteries. And, lastly, I had used it with perfect success in the living human subject, in shutting up the arteries that were divided in three cases of excision of the cancerous mamma. In the last of these three cases I had to close six separate arteries with an equal number of acupressure needles. But, not being a practical surgeon, I had, of course, no opportunity of testing it myself in any of the graver operations of surgery, such as amputations of the limbs. Through the kindness, however, of my surgical friends, I am able to report the effects of acupressure in four cases of amputation in which it has been employed during the past month.

For the first application of acupressure to the arrestment of hemorrhage after amputation of the limbs, I am indebted to one of the most accomplished and advanced surgeons in this country—my friend, Dr. Greig, of Dundee. Formerly as surgeon to our army in the East, and latterly as surgeon to the large hospital at Dundee, Dr. Greig has enjoyed extensive opportunities as an operator. His very interesting letters to me on the subject of acupressure indicate the change and struggle which every earnest and ingenuous mind has in setting aside old-established and cherished practices for the adoption of what is new. When I first took the liberty of directing his attention to the subject, as contained in an abstract of a paper on it in the January number of the *Edinburgh Medical Journal*, and asked him to be so good as to test the plan, he wrote me, January 8, 1860, that he could "see no great difficulty in giving the thing a fair trial." But he adds: "Of its general adoption I have great doubts. We have been always taught to look upon the ligature as the only true means of arresting hemorrhage, and this feeling it is somewhat difficult to get out of one's mind. Your illustration of fixing a flower in the lapelle of the coat by means of a pin, explains the whole thing."

*Case* 1.—Two days afterwards, January tenth, Dr. Greig wrote me: "I performed amputation at the forearm, this afternoon, in a case of laceration of the hand from the bursting of a gun, and I used the needles instead of ligatures for arresting hemorrhage. Both the radial and ulnar arteries bled freely, but were easily controlled by a needle placed on each, almost half an inch above the cut end. Both needles were, of course, in the palmar or anterior flap, and were applied *quite as easily as a ligature*." These last words are underscored in Dr. Greig's letter, and show that thus the very first trial of acupressure proved as easy as deligation in the hands of a surgeon who for years had been in the constant practice and habit of applying ligatures to arteries for the stanching of hemorrhage in his operations.

*Case* 2.—Three days afterwards, January thirteenth, Dr. Greig again wrote as follows: "I have had another amputation at the middle of the forearm to-day, and used acupressure with ease and success. The process, so far as I have tried it, is *the simplest* that one can imagine; and, unless I see some good reason for changing my mind, it must ultimately come into universal adoption. It is really surprising how very little pressure is required to stop bleeding from an artery. In fact, I had no idea of it till I tried acupressure."

On January twentieth, Dr. Greig writes: "Both the cases of amputation in which I used acupressure have done remarkably well. There has been less irritation and less suppuration, and the wounds are healing more kindly than had ligatures been employed. The first case did not close by the first intention, owing to part of the anterior flap having been lacerated by the explosion. The second has gone on as well as could be wished—no fever, no irritation—and the wound is healing by the first intention. What surprises me more than anything else is the very small amount of pressure which is required to stop arterial hemorrhage. In passing the needle over an artery I do not think it will be found necessary to turn it sharply over the vessel, thereby binding it very tightly to the flap. Such a degree of pressure is by no means required. Less irritation is caused by passing the needle more lightly across the artery, and taking in more tissue along with it."

"It is a great comfort also," Dr. Greig adds, "to both patient and surgeon, that by acupressure the artery is closed in about forty-eight hours (a large artery may, of course, require a longer time,) and all cause of irritation at once removed. In my first case I allowed the needles to remain in for three days; but in future I will consider two days long enough; and, for all I know, perhaps it is longer than is required."

"I have now the greatest faith in acupressure. I intend employing it in all kinds of cases that may come under my care, and I will have no fear whatever to use it in my first thigh amputation."

"In giving directions for securing the vessel, you advise the surgeon to place the fore finger over its bleeding mouth, etc. Now you will find it much better when you have a flap to keep the finger of the left hand on the skin side and use the thumb. You feel the vessel beating between the thumb and the fore finger, and you can introduce the needle in the dark."

I heard again from Dr. Greig, on January twenty-third: "The amputations," he states, "are doing well, and both patients are walking about the wards. Yesterday," he continues, "at a case of removal of the mamma, I again used the needles, and easily arrested the hemorrhage from two arterial branches which were spouting freely in the upper or axillary flap. A small branch of an intercostal was the only other bleeding vessel, and torsion was used for it. Nothing could have been easier or more beautiful than acupressure applied in this case, as the procedure was seen in its simplest form—more so than in a flap. I see," Dr. Greig adds, "that in France, M. Foucher (see *Medical Times and Gazette* for January 21st, p. 72,) has tried acupressure on the dead subject, and also on a dog. I wonder why he did not try it in an amputation. Nothing can be easier; and if a surgeon uses it once I am sure he will do so again."

*Case* 3.—On January thirtieth, I had an opportunity kindly afforded me by Mr. Edwards, lecturer on surgery here, of applying acupressure to stop the bleeding following an amputation of the foot through the first row of tarsal bones. The patient had been unable to work for one or two years, in consequence of an injury to the foot, which led to necrosis, and intractable caries of the anterior row of tarsal bones. He was a strumous subject, and his health was much damaged and broken down by the effects of the disease. Mr. Edwards performed the operation with great dexterity and rapidity, and the four or five vessels that bled were easily secured by as many acupressure needles. The section of the astragalus showed the existence of some disease in its cancellated tissue, which necessitated the removal with the gouge of a portion of its structure. The whole surface of the bone laid bare by the saw and gouge was vascular, and continued to ooze out blood as long as it was exposed. But as the patient was very weak and reduced, Mr. Edwards was anxious to close and stitch up the wound as soon as possible, and before the chloroform-sleep was over. The needles were all removed from the stump about fifty hours after the amputation. During the two subsequent days, there twice occurred a slight oozing of blood from the outer angle of the wound, but not more than enough to redden the moist dressings; and this altogether ceased on the removal of an old ash-colored clot from the situation above mentioned. To-day (eight days from the date of the operation) the stump is healing kindly, and the patient feels well.

*Case* 4.—In the preceding three cases of amputation, acupressure was effected by passing the needles from the cutaneous surface of the flap, over the track of the bleeding vessel, and then causing their points to emerge through the skin at some distance. In other words, in all these cases the cutaneous portion of the flap was used as the point of resistance against which the wounded artery was compressed by the bridge of the needle passing over it. In an instance of amputation of the leg immediately below the knee, performed on January thirty-first, in the hospital at Carlisle, by my esteemed friend Mr. Page, I had an opportunity of applying acupressure in another of the modes suggested in my paper on the subject, viz., by compressing the principal bleeding arteries against a neighboring bone as the resistant point. The cause leading to the amputation was very extensive, old-standing, irremediable disease of the tibia. It is, I believe, generally acknowledged among surgeons, that in consequence of the deep situation of the two tibial arteries, between the tibia and fibula, and in proximity to the interosseous ligament, seizure and deligation of these vessels in amputation immediately below the knee are, as a general rule, more difficult to accomplish than the ligature of the arteries cut across in any of the other amputations of the limbs.

After Mr. Page had removed the diseased limb in the case in question, I stayed the hemorrhage from the two tibial arteries by compressing and closing them with two needles introduced through the cutaneous surface of the anterior flap, about half an inch above the level of the ends of the amputated bones. The points of these needles, after producing the requisite degree of compression of the vessels against the bone, were pushed onward into the substance of the stump behind. They were not, in this way, visible at any point on the raw surface of the stump. The first needle that I passed failed in producing an adequate degree of compression; but the two next succeeded. Half-way down on the inner surface of the large and fleshy posterior flap, an artery gave rise to some difficulty, for a reason which I had not previously been prepared for. I passed a needle through the flap, a few lines on the upper or cardiac side of this bleeding orifice, so as to produce a sufficient degree of compression across the supposed track of the vessel leading to it, but without the effect of arresting the hemorrhage. On sponging the bleeding point, and examining it more carefully, we found that the jet from the artery was coming from below upward, and not from above downward. In consequence of this discovery, I removed the acupressure needle, passed it through the flap nearer its apex, so as to produce compression two or three lines *below* instead of above the bleeding point, (on the peripheral instead of the cardiac side of that point,) and the hemorrhage was forthwith arrested. Mr. Page closed the wound most carefully with a large number of metallic sutures. He withdrew the acupressure needles seventy-one hours after their introduction. In a letter which I received from him four days after the operation, Mr. Page says: "The man continues to eat and sleep well. Indeed," he adds, "I never had a patient who suffered less from amputation of the leg; and the condition both of the patient and the stump are altogether most satisfactory."

In addition to the four preceding larger amputations, I have heard of some smaller amputations about the fingers and hands performed during the last month, in which acupressure was successfully used for arresting the subsequent hemorrhage. I saw a case of amputation of one of the fingers, in which my pupil, Mr. Pierce Simpson, operated. The arterial bleeding, as well as some general oozing from the surface of the flap, ceased immediately upon the introduction of an acupressure needle. The finger was irritated, and its vessels full and injected, in consequence of the effects of a severe injury received two weeks previously.

In one of the extracts from Dr. Greig's letters, the facility of the arrestment of arterial hemorrhage by acupressure, as compared with the ligature, is adverted to. Any surgeon accustomed to try both will speedily find acupressure to be far the easier and the more expeditious process of the two. Besides, in applying ligatures, the operator always requires the aid of an assistant; he can himself, however, apply acupressure needles without any such aid. While acupressure is thus far simpler in its application, it will also, I firmly believe, be found also far surer in its results as regards all the chances of obtaining complete reunion of the sides of the flaps by the first intention; and far safer, too, as respects the avoidance of the dire mischances of surgical fever from the absorption of morbid and septic matters formed by local suppurations and sloughs in the depth of the wound. Every ligature properly applied around the isolated extremity of a bleeding artery invariably tears and mechanically lacerates the two interior coats of the artery, and afterwards as invariably produces ulceration, suppuration, and local gangrene at the tied point, before the thread can possibly separate and become detached. No such severe and morbid local consequences necessarily result when a bleeding vessel is closed by the temporary contact of a needle; for acupressure does not involve any laceration, ulceration, or mortification of the arteries, which it occludes.

EDINBURGH, *6th February*, 1860.

*Editorial Note on Acupressure, from the Medical Times and Gazette*, March 17, 1860.—Acupressure is making progress. There are now known in Edinburgh eleven amputations of the limbs in which the bleeding was arrested by means of acupressure, viz., two of the thigh; four of the leg; one of the arm; and four of the forearm.

Besides these there are two amputations of the ankle-joint or foot; several amputations of the toes and fingers; and various instances of excision of the mamma, etc. All the cases have recovered, or are in the way of recovery, and secondary hemorrhage appears to have taken place in none. The first case in which acupressure was used after amputation of the thigh was in a patient who had spreading gangrene of the leg as the result of a machinery accident. Dr. James Struthers, of Leith, amputated the limb, and removed the needle which secured the femoral artery on the fourth day. In the other thigh amputation, in the practice of Mr. Crompton, of Birmingham, the acupressure needle was withdrawn in fifty-two hours. Our Paris correspondent writes as follows: "M. Foucher, who has just been appointed to the Necker, removed a tumor from the neck of a woman two days ago. He was prepared to employ acupressure, but, with the exception of a small artery, no vessel of any consequence was wounded. This vessel was perfectly controlled by Simpson's method; the needle employed, which was substituted for a ligature, having been removed toward the termination of the dissection, and before the wound was dressed. Foucher is quite disposed to give acupressure a fair trial, and waits only a favorable opportunity to employ it in an amputation. He is pretty confident that it will ultimately supersede the ligature."

2. INTERMITTENT LAMENESS IN THE HORSE FROM EMBOLI, OR OBLITERATION OF THE ILIAC ARTERIES. By *Wörz.*—Wörz adds two observations to twenty-seven others collected by Hering, as recorded by himself in the "Repertorium." The first of the two cases now published attacked a troop-mare of the royal body-guard, five years old. It is not substantiated by a post-mortem examination, inasmuch as, after repeated attacks of lameness, the mare was cast. In addition to the lameness, there were symptoms of paralysis of the hind legs.

The next case occurred in a bay gelding, sixteen years old, a cross between Arab and English blood.

This horse seems long to have suffered from weakness in the hind extremities. About the middle of July, 1857, the debility and awkward gait were marked, but the horse appeared otherwise healthy, and was exercised daily, walking for about half or three quarters of an hour. On the 25th of August, while walking, lameness in the off hind leg became manifest. He was moved with difficulty into the stable, where he fell on the straw, and held the above-mentioned limb out stiff. The breathing was labored, and nares dilated. In about a quarter of an hour, the horse rose and eat, and for ten days remained quiet, and apparently recovering.

On the 5th of September, he was for the first time taken out about half-past eight in the morning, and saddled. In about two minutes, the rider observed that the horse was dropping behind, and, in addition to stiffness of the off hind leg, the near hind one became paralyzed, and apparently very painful; sweat broke out all over the animal's body, and breathing was very labored. On being taken into the stable, the horse fell, in a perfectly helpless manner; but in about half an hour rose again. The near leg was useless, and no weight could be borne upon it. It was cold, and deprived of sensibility. The pulse was small, and 60. On examination per rectum, the end of the aorta was felt to be hard and thick, and the iliac arteries not pulsating. On the 6th of September, the pulse was 60, and small; appetite slight; and the near hind leg still stiff and cold. On the 7th, there was an increase of pain, and labored breathing; and as the diagnosis of arterial obliteration was so clear, and recovery impossible, the horse was destroyed, and a post-mortem examination performed, in the presence of Professor Hering. This revealed plugging of aorta, and external and internal iliac arteries; the right crural had, however, remained free.—(*Edinburgh Veterinary Review*, January, 1860.)

